# Supplementary material for: Clinical features, antimicrobial susceptibility patterns and genomics of bacteria causing neonatal sepsis in a children’s hospital in Vietnam: protocol for a prospective observational study
Source: BMJ Open. 2018 Jan 24;8(1):e019611. doi: 10.1136/bmjopen-2017-019611 (PMC5786094; doi:10.1136/bmjopen-2017-019611)
Supplement: Author's manuscript [file bmjopen-2017-019611.draft_revisions.pdf]

BMJ Open

BMJ Open is committed to open peer review. As part of this commitment we make the peer review history of every article we publish publicly available.

When an article is published we post the peer reviewers' comments and the authors' responses online. We also post the versions of the paper that were used during peer review. These are the versions that the peer review comments apply to.

The versions of the paper that follow are the versions that were submitted during the peer review process. They are not the versions of record or the final published versions. They should not be cited or distributed as the published version of this manuscript.

BMJ Open is an open access journal and the full, final, typeset and author-corrected version of record of the manuscript is available on our site with no access controls, subscription charges or pay-per-view fees (http://bmjopen.bmj.com).

If you have any questions on BMJ Open's open peer review process please email editorial.bmjopen@bmj.com

BMJ Open

The clinical features, antimicrobial susceptibility patterns, and genomics of bacteria causing neonatal sepsis in a children's hospital in Vietnam: protocol for a prospective observational study

Journal:	*BMJ Open*
Manuscript ID	bmjopen-2017-019611
Article Type:	Protocol
Date Submitted by the Author:	15-Sep-2017
Complete List of Authors:	Toan, Nguyen; Oxford University Clinical Research Unit (OUCRU), Enteric Group<br>Darton, Thomas; Hospital for Tropical Diseases, Wellcome Trust Major Overseas Programme, Oxford University Clinical Research Unit<br>Boinett, Christine; Hospital for Tropical Diseases, Wellcome Trust Major Overseas Programme, Oxford University Clinical Research Unit<br>Campbell, James; Hospital for Tropical Diseases, Wellcome Trust Major Overseas Programme, Oxford University Clinical Research Unit<br>Karkey, Abhilasha; Hospital for Tropical Diseases, Wellcome Trust Major Overseas Programme, Oxford University Clinical Research Unit<br>Kestelyn, Evelyne; Hospital for Tropical Diseases, Wellcome Trust Major Overseas Programme, Oxford University Clinical Research Unit<br>Thinh, Le; Children's Hospital 1<br>Mau, Nguyen; Children's Hospital 1<br>Tam, Pham Thi Thanh; Children's Hospital 1<br>Nhan, Le Nguyen Thanh; Children's Hospital 1<br>Minh, Ngo Ngoc Quang; Children's Hospital 1<br>Phuong, Cam; Hanh Phuc International Hospital<br>Hung, Nguyen Thanh; Children's hospital one<br>Xuan, Ngo; Pham Ngoc Thach University of Medicine<br>Thuong, Tang; Department of Health<br>Baker, Stephen; Oxford University Clinical Research Unit, Enteric infections
Keywords:	Sepsis, Neonates, Clinical features, Antimicrobial resistance, Genomics, Outcomes

SCHOLARONE™
Manuscripts

The clinical features, antimicrobial susceptibility patterns, and genomics of bacteria causing neonatal sepsis in a children's hospital in Vietnam: protocol for a prospective observational study

Nguyen Duc Toan [1,2,3,4], Thomas C. Darton [1,5], Christine J. Boinett [1], James I. Campbell [1], Abhilasha Karkey [1], Evelyne Kestelyn [1,2], Le Quoc Thinh [3], Nguyen Kien Mau [3], Pham Thi Thanh Tam [3], Le Nguyen Thanh Nhan [1,3], Ngo Ngoc Quang Minh [3], Cam Ngoc Phuong [6], Nguyen Thanh Hung [3,4], Ngo Minh Xuan [4], Tang Chi Thuong [4,7], Stephen Baker [1,2,8]

[1] Hospital for Tropical Diseases, Wellcome Trust Major Overseas Programme, Oxford University Clinical Research Unit, Ho Chi Minh City, Vietnam

[2] Centre for Tropical Medicine and Global Health, Nuffield Department of Clinical Medicine, University of Oxford, Oxford, United Kingdom

[3] Children's Hospital 1, Ho Chi Minh City, Vietnam

[4] Pham Ngoc Thach University of Medicine, Ho Chi Minh City, Vietnam

[5] Department of Infection, Immunity and Cardiovascular Disease, University of Sheffield Medical School, Sheffield, United Kingdom

[6] Hanh Phuc International Hospital, Binh Duong Province, Vietnam

[7] Department of Health, Ho Chi Minh City, Vietnam

[8] Department of Medicine, University of Cambridge, Cambridge, United Kingdom

Corresponding author: Professor Stephen Baker, Hospital for Tropical Diseases, 764 Vo Van Kiet, District 5, Ho Chi Minh City, Vietnam. Tel: 84-28-3923 7954. sbaker@oucru.org

Running title: Vietnam neonatal sepsis protocol

For peer review only

Abstract

Introduction: The clinical syndrome of neonatal sepsis, comprising signs of infection, septic shock, and organ dysfunction in infants ≤4weeks of age, is a frequent sequel to bloodstream infection and mandates urgent antimicrobial therapy. Bacterial characterisation and antimicrobial susceptibility testing is vital for ensuring appropriate therapy, as high rates of antimicrobial resistance (AMR), especially in low and middle-income countries (LMICs), may adversely affect outcome. Ho Chi Minh City (HCMC) in Vietnam is a rapidly expanding city in Southeast Asia with a current population of almost 8 million. There are limited contemporary data on the causes of neonatal sepsis in Vietnam, and we hypothesize, that the emergence of multi-drug resistant bacteria is an increasing problem for the appropriate management of sepsis cases. In this study, we aim to investigate the major causes of neonatal sepsis and assess disease outcomes by clinical features, antimicrobial susceptibility profiles, and genome composition.

Method and analysis: We will conduct a prospective observational study to characterize the clinical and microbiological features of neonatal sepsis in a major children's hospital in HCMC. All bacteria isolated from blood subjected to whole genome sequencing. We will compare clinical variables and outcome between different bacterial species, genome composition and AMR gene content. AMR gene content will be assessed and stratified by species, year, and contributing hospital department. Genome sequences will be analysed to investigate phylogenetic relationships.

Ethics and dissemination: The study will be conducted in accordance with the principles of the Declaration of Helsinki and the International Council on Harmonization Guidelines for Good Clinical Practice. Ethics approval has been provided by the Oxford Tropical Research Ethics Committee and Children's Hospital 1. The findings will be disseminated at international conferences and peer-reviewed journals.

Trial registration: ISRCTN69124914

Ethics references: Oxford (Oxford Tropical Research Ethics Committee 35-16), Vietnam (Children's Hospital 1 Ethics Committee 73/GCN/BVND1)

For peer review only

Strengths and limitations of this study

- Little is known about the current aetiological agents of neonatal sepsis in Vietnam; the clinical and microbiological information generated by this study will inform local clinical practice and guidelines.
- This prospective study aims to integrate clinical assessments with microbiological and detailed whole genome sequence data to accurately and comprehensively characterize the aetiology and outcome of neonatal sepsis in this high mortality setting.
- Limitations to the study include ethical issues involved in collecting samples from severely ill neonates, lack of systematic guidelines ensuring accurate and transferable definitions of clinical phenotype are used, stochastic variations in numbers of cases collected due to seasonal variation and continuous changes in community antimicrobial and vaccine use.
- This study is being performed at the largest secondary/tertiary paediatrics centre in Southern Vietnam, data collection at a single site may limit the applicability to other hospitals in the country.

For peer review only

Background

Neonatal sepsis is widely recognized as a clinical syndrome of systemic inflammation in response to, or occurring the same time as, a possible or proven infection (frequently by identifying bacterial bloodstream infection) occurring in children ≤28 days of age. A consensus definition of neonatal sepsis has remained a challenge.(1) Globally, the incidence of neonatal sepsis is estimated to be 1–5 cases/1,000 live births but is lower in full-term neonates (1–2 cases/1,000 live births), in whom the incidence is higher in males than females.(2,3) Early-onset sepsis is defined as the start of sepsis symptoms within 72 hours of birth,(1,4) and is often caused by vertical transmission of pathogens during delivery as a result of chorioamnionitis or maternal genital tract colonization.(5) Late-onset sepsis, occurring after 72 hours from birth,(1,6) may be caused by similar vertical transmission or horizontal transmission mechanisms, due to direct contact with the surrounding environment, attendant healthcare staff, or any invasive procedures.(7)

Neonatal sepsis in Southeast Asia

Neonatal sepsis remains a leading cause of neonatal hospital admission, morbidity, and mortality in lower and middle income countries (LMICs).(8) In this setting, bacterial infection, including bacteraemia, is complicated by multidrug resistance, particularly related to healthcare acquired infection, and effective management of neonatal sepsis is increasingly problematic.(8) Recently, the World Health Organization (WHO) has acknowledged the problem of AMR as an endemic and widespread problem in LMICs.(9) In many LMICs untreatable bacterial infections with broadly AMR pathogens are no longer a threat but a common reality. AMR in LMICs represents one of the biggest threats to global health, and are one of the greatest current challenges in infectious disease research.

While AMR is an issue with all types of bacterial infection, the issue is most acute in management of clinical sepsis. This is a particular problem in neonates, due to high mortality/morbidity rates and the timely need for rapid detection and treatment of the causative pathogen. Sepsis demonstrates extensive geographical diversity in both aetiology and proportions of AMR bacteria isolated.(10,11) Understanding the local and regional epidemiology of sepsis in hospitalized neonates is crucial in the

For peer review only

development of rational management and treatment guidelines, especially in high-risk AMR LMICs locations like Vietnam.

Sepsis and AMR in Vietnam

Bacterial sepsis is classified into two major groups according to place of acquisition. Hospital-acquired sepsis is defined in patients with clinical manifestations of sepsis and a confirmatory blood culture collected >48 hours following hospital admission.(12) Hospital-acquired sepsis is a major threat to patient safety, and in locations with poor surveillance and infection control programs such infections are associated with high mortality rates. The incidence of AMR bloodstream infections in Vietnam has increased over recent years, and is predicted to increase further.(13) This trend has comprised an increase in both Gram-negative and Gram-positive pathogens, chiefly *Escherichia coli, Klebsiella pneumoniae, Acinetobacter baumannii, Staphylococcus aureus,* and enterococci.

Community-acquired sepsis is also an important cause of fever among patients admitted to hospitals across South and Southeast Asia. In our practice, community-acquired sepsis is defined as the presence of sepsis with a confirmatory blood culture collected within 48 hours after hospital admission.(14) Distinguishing bacterial infections from other common causes of fever, such as malaria or dengue, can be challenging without diagnostic laboratory support.(15) Important causes of community-acquired bloodstream infection (CA-BSI) in LMICs include *Salmonella* serovars Typhi and Paratyphi A, *E. coli*, *K. pneumoniae*, *S. aureus* and *S. pneumoniae*. We have reported increasing levels of AMR in these common community-acquired pathogens and highlighted the difficulties of accurate diagnosis with traditionally available diagnostics. For example in Nepal and Vietnam antimicrobial resistance in *Salmonella* Typhi and *Salmonella* Paratyphi A has severely restricted the options available for antimicrobial treatment.(16)

The changing aetiology of BSI in Vietnam

The aetiology of CA-BSI in Vietnam has changed considerably over the 20 years. A previous study documented the decline of *Salmonella* Typhi from 2002, the predominant pathogen until this point,

For peer review only

and the subsequent increase in non-typhoidal *Salmonella* and other opportunistic HIV-associated pathogens.(17) This shift is likely to reflect a changing landscape of infectious disease related to the HIV epidemic, urbanization, and secondary social determinants within Vietnam. Vietnam, as with many countries in Asia, is undergoing a rapid economic transition, and programmes to improve sanitary conditions have reduced the overall risk of water-borne infections. HIV-associated opportunistic pathogens have now emerged as the leading cause of bloodstream infections and the primary cause of mortality in hospitalized adult patients in this location. These studies were performed at the Hospital for Tropical diseases in Ho Chi Minh City and thus included mainly adults, including those with HIV, and children but not neonates.(18–20) Therefore, these observations may not be fully representative of the situation in neonates.

Knowledge gaps

Little is known about contemporary antimicrobial susceptibility patterns and their underlying genetic determinants in the major causes of neonatal bacterial of sepsis in Vietnam. Furthermore, the impact of antimicrobial susceptibility and other virulence factors on disease progression and outcome in neonates in LMICs is also not well documented. To address these issues, we aim to investigate the aetiology of pathogens associated with bacterial sepsis in neonates and to detail the effects of reduced antimicrobial susceptibility on the outcome of sepsis in neonates. This will be a clinical and microbiology laboratory research project between Children's Hospital 1 and the Oxford University Clinical Research Unit in Vietnam. This study will be a conduit for introducing molecular biology for bacteriology into routine hospital care this children's hospital and will lead to future studies investigating appropriate empirical treatment for bacteraemia and the impact of antimicrobial resistance on the outcome of sepsis in the paediatric and neonatal population in Vietnam.

Rationale, aim and objectives

To understand the causes of neonatal sepsis and to best inform antimicrobial treatment regimes in our setting we will perform a prospective observational study at Children's Hospital 1 in Vietnam from 2017 to 2019. There are limited contemporary data on the causes of bacterial sepsis in neonates in

For peer review only

Vietnam. We hypothesize that there have been recent increases in multi-drug resistant Gram-negative bacteria causing sepsis in this high-risk group, and that methicillin-resistant *Staphylococcus aureus* (MRSA) has emerged as an important pathogen. We further aim to investigate the clinical features, major causes of neonatal sepsis, and the distribution of pathogens by department, their antimicrobial susceptibility patterns and the genomic profiles of the isolated bacteria as well as their association with disease outcomes.

Primary objectives

- To describe the clinical characteristics of neonates with sepsis, including community and hospital-acquired sepsis, early and late-onset sepsis.
- To determine the aetiology of neonatal sepsis and the distribution of pathogens by clinical department including the Neonatology Department and the Neonatal Intensive Care Unit.
- To determine the antimicrobial susceptibility profiles of the bacteria causing neonatal sepsis and the AMR profiles occurring in community and hospital-acquired infections.
- To analyse the impact of specific bacteria and AMR profile on the outcomes (mortality, length of stay and cost of treatment) of neonates with sepsis.
- To determine the genome sequences of bacterial strains associated with neonatal sepsis

Secondary objectives

- To determine the AMR profiles and gene distribution of isolated bacteria by clinical departments to add insight into the circulation of bacteria associated with hospital acquired infections.
- To study the genes catalysing resistance to antimicrobials commonly used to treat neonatal sepsis (specifically third/fourth generation cephalosporins, fluoroquinolones and carbapenems).

For peer review only

Methods

Study design

This protocol describes a prospective, non-interventional, observational study to characterize the clinical features of neonates with sepsis at Children's Hospital 1 in Ho Chi Minh City in Vietnam between 2017 and 2019, the microbial population structure, antimicrobial susceptibility patterns and the AMR genes of the bacteria causing that sepsis. All organisms isolated from blood will be stored and archived for molecular characterization.

Study sites

Children's Hospital 1 (Neonatology Department, Neonatal Intensive Care Unit, Microbiology Department) in Ho Chi Minh City in Vietnam. The estimated population of the city was 8.4 million in 2016, and 23.8% are children 0-14 years of age.(21) Children's Hospital 1 is the largest tertiary paediatrics centre in Southern Vietnam with 1,400 inpatient beds and >1,600 staff members. The hospital receives ~1.5 million outpatient visits and 95,000 admissions each year. Care is provided to all children <15 years old from Ho Chi Minh City and other provinces of Southern Vietnam. The neonatal centre at CH1 currently has 120 inpatient beds for the neonatology department and additional 30 beds in the neonatal intensive care unit. The overall mean rate of positive blood cultures in our hospital is 7% per year.

Definitions

Definition of sepsis

A sepsis episode in this study is defined as isolation of a clinically relevant pathogen from ≥1 blood culture, drawn from a neonate with ≥1 clinical or laboratory sign of sepsis (Table 1).(22)

Diagnosis of neonatal sepsis

Systematic guidelines concerning which patients should have blood cultures performed are not strictly defined in our hospital, although blood culture results are used to confirm the diagnosis of sepsis in neonates with a compatible clinical presentation. We use the criteria suggested by the European

For peer review only

Medicines Agency (EMA) in 2010 for the diagnosis of "probable sepsis" and "confirmed sepsis" in neonates:(22)

- Probable sepsis: ≥2 clinical and ≥2 laboratory signs.
- Confirmed sepsis: ≥1 positive culture of a pathogen and ≥1 clinical or laboratory sign.

Sample size

In this prospective observational study, we aim to recruit all patients with available data who fulfil the inclusion criteria and are admitted to Children's Hospital 1 in Ho Chi Minh City in Vietnam from 2017 – 2019. Based on retrospective surveillance data, we estimate recruitment of 800 participants during the study period. Blood cultures will be performed in all cases, we expect to yield ~400 bacterial isolates.

Participant selection and recruitment

Inclusion criteria

Neonates (≤1 month of age) with a diagnosis of "probable" or "confirmed" sepsis who have had a blood culture taken and who are an in-patient at Children's Hospital 1 will be recruited into the study, after written informed consent has been given by a parent or guardian.

Exclusion criteria

Patients will be excluded when informed consent is not provided, the length of hospital stay less than 24 hours, imminent and inevitable death, or the patient has been previously recruited in the study.

Identification of participants

All doctors and nurses in the Department of Neonatology, Neonatal Intensive Care Unit and Microbiology Department of the study hospital will be informed about, and trained for, this clinical investigation. In addition, those working in the microbiology department, neonatology department, and neonatal intensive care unit will also be informed and involved in the study. These staff will be trained to identify eligible patients and how to notify investigators.

Informed consent

For peer review only

Trained, GCP-accredited, members of the study team will collect informed consent. The team will discuss the study with the accompanying parent/guardian, or, if both parents are deceased or not actively involved in child care, the main long-term carer of the child will be accepted as the guardian and considered able to give consent for the study. Study staff will describe the purpose of the study, the study procedures, possible risks/benefits, the rights and responsibilities of participants, and alternatives to enrolment. The parent/guardian will be invited to ask questions, which will be addressed by study staff, and they will be provided with appropriate contact numbers if they have any subsequent questions. If the parent/guardian agrees for the child to participate, they will be asked to sign and date an informed consent form. A copy of the patient information sheet and the informed consent form will be given to them to keep. In addition to the procedures above, illiterate signatories will have the Informed Consent Form read to them in the presence of a witness who will sign to confirm this. All patient information sheets and consent forms will be written in the local language and will use terms that are easily understandable.

Study procedures

An investigator will routinely record and collect demographic, clinical and laboratory information of the patients, the date of blood draw, the number of blood culture bottles inoculated, the result of the culture (whether positive or negative) and the susceptibility of the isolate to commonly used antimicrobials. Data from these records will be subsequently entered into CliRes Data Management System of Oxford University Clinical Research Unit. These will be source data for this study. The number of patients admitted to the hospital annually will be obtained from hospital records. As part of this study we request that all isolates from blood are stored and archived at –80^{0}C. These isolates will be re-cultured and the identification will be re-confirmed. Selected isolated organisms from blood will have further molecular characterization at a later date.

Data collection

Demographic and clinical assessments

For peer review only

Data on neonatal sepsis at Children's Hospital 1 will be collected to the case report form. These data will include administrative data, demographic data, clinical characteristics, laboratory results, diagnoses, treatments and outcomes. The Neonatal Therapeutic Intervention Scoring System (NTISS) will be used to estimate the disease severity.(23)

Microbiological assessments

Available routine microbiology data on neonatal bloodstream infections Children's Hospital 1. These data will include pathogenic agents isolated from blood culture and antimicrobial susceptibility profile of isolated bacteria (routine panel of antimicrobials). Selected isolates will have additional antimicrobial susceptibility testing, molecular analysis for antimicrobial resistance genes and genome sequencing of selected strains defined by antimicrobial susceptibility data.

Laboratory methods

Microbiology testing

When required for checking or to non-routine antimicrobials, antimicrobial susceptibility testing of the pathogens isolated will be performed by disk diffusion using guidelines established by the Clinical and Laboratory Standards Institute (CLSI) and, when required, by minimum inhibitory concentration estimation (MIC) using the VITEK 2 COMPACT automated machine. Antimicrobial susceptibilities tested will include nalidixic acid, ciprofloxacin, ceftriaxone, cefepime, ampicillin, trimethoprim-sulfamethoxazole, azithromycin, imipenem, colistin and amikacin for all Gram-negative organisms and oxacillin and vancomycin in Gram-positive organisms. The production of extended-spectrum beta lactamases (ESBL) will be investigated using the double-disc synergy test by comparing zone sizes between ceftazidime discs against ceftazidime-clavulanic acid discs and cefotaxime discs against cefotaxime-clavulanic acid discs. Isolates with an increase in diameter of inhibitory zone of equal to or more than 5 mm by the synergy of clavulanate will be considered ESBL positive.

Bacterial storage

Organisms will be sub-cultured onto 5% blood agar and the purity of the isolate will be tested before storage in 20% glycerol at –80°C.

For peer review only

Isolation of nucleic acids

Isolates will be re-cultured and their identification re-checked. DNA will be extracted from bacterial isolates using the Wizard Genomic DNA Extraction Kit (Promega, Fitchburg, USA). The quality and concentration of the DNA will be assessed using a nano-drop spectrophotometer prior to PCR amplification and the Quant-IT Kit (Invitrogen, Carlsbad, CA) prior to DNA sequencing.

PCR for resistance genes

The primary focus study is to investigate the distribution of antimicrobial resistance genes in bacteria causing neonatal sepsis. Therefore, all Gram-negative organisms will be investigated by PCR to detect genes catalyzing resistance to cephalosporins, fluoroquinolones and carbapenems. Conventional PCR will be performed for the following classes of resistance genes using previously described methods. The multiplex and monoplex PCRs are described in these publications. This panel of PCRs will be used; PCR1 - AmpC (MOX-1, MOX-2, CMY-1, CMY8-11), PCR2 - AmpC (LAT-1 to LAT-4, CMY2-7, BIL-1), PCR3 - AmpC (DHA1 and DHA-2), PCR4 - AmpC (ACC), PCR5 - AmpC (MIR-1T, ACT-1), PCR6 - AmpC (FOX-1-5b), PCR7 - ESBL (CTX-M1), PCR8 - ESBL (CTX-M2), PCR9 - ESBL (CTX-M9), PCR10 - ESBL (CTX-M8/M25), PCR11 - ESBL (TEM), PCR12 - ESBL (SHV), PCR13 - ESBL (OXA1, 4, 30), PCR13 - qnrA, B, S, and PCR13 - gyrA, B, C, PCR14 - NDM-1, PCR 15 - mecA/Van.(11,24–29)

Genome sequencing

Selected organisms (on the basis of their susceptibility profiles and resistance gene content) will be genome sequenced. We aim to sequence the greatest cross-section of organism groups as possible (i.e. all *Staphylococci* or all *Klebsiella*). Selected bacterial isolates will be sequenced at Oxford University Clinical Research Unit in Vietnam. Briefly, index-tagged paired end Illumina sequencing libraries will be prepared using one of 96 unique indexing tags as previously described. These will be combined into pools of uniquely tagged libraries and sequenced on the Illumina Genome Analyzer or

For peer review only

HiSeq sequencer according to manufacturer's protocols to generate tagged 54-100 bp paired-end reads. This is a previously described for Gram-negative organisms and *Staphylococcus*.(20,30,31)

Analysis plan

Statistical comparisons

Data will be presented in the form of tables and bar charts for descriptive variables i.e. number of organisms per year and number of AMR organisms per year. Statistical comparisons of features between groups (positive/negative blood culture, Gram negative/Gram positive bacteria, survival/non-survival etc.). Time trend analysis of the cultured isolates by month and the antimicrobial susceptibility patterns will be determined. All statistical analysis will be performed using Stata version 14 (Stata Corp LP, College Station, TX, USA) and R. P-values of ≤ 0.05 will be considered significant.

Antimicrobial resistance genes and genome sequencing

The presence/absence of antimicrobial resistance genes will be reported as proportions per organism and then stratified by organism, year, and hospital department. Genome sequences will be determined to study phylogenetic relationships, the presence/absence of virulence genes and also AMR gene content and firstly analysed by species and then group by their Gram-stain results. Briefly, for phylogenetic analysis, chromosomal Single Nucleotide Polymorphism (SNP) alleles will be concatenated for each strain to generate a multiple alignment of all SNPs. For maximum likelihood (ML) analysis, RAxML will be run using the generalized time-reversible model and one thousand bootstrap pseudo-replicate analyses were performed to assess support for the ML phylogeny. Root-to-tip branches will be extracted from the ML tree using the program TreeStat. The relationship between root-to-tip distances and year of isolation will be analyzed using linear regression. For BEAST analysis (v1.6), a GTR+Γ substitution model and defined tip dates, as the date of isolation will be used.(30–32) To detect the presence or absence of genes read sets will be assembled using the *de novo* short read assembler Velvet and Velvet Optimizer. Organism specific read sets will then be aligned to

For peer review only

the pan-genome. Taxonomic investigation of accessory and AMR genes will be performed using MG-RAST v3.2.

Ethics, regulatory approvals and governance

This study is sponsored by the University of Oxford and will be monitored by the Clinical Trials Unit at Oxford University Clinical Research Unit (OUCRU). The Principal Investigator (SB) will ensure that this study is conducted in accordance with the principles of the Declaration of Helsinki and the terms of approval of the appropriate ethical committees.(33) The study will be conducted in full conformity with relevant regulations and with the International Council on Harmonization (ICH) Guidelines for Good Clinical Practice (GCP).(34) This protocol and the relevant supporting document have already had the approvals of the Oxford Tropical Research Ethics Committee (OxTREC) and the institutional review board (IRB) of Children's Hospital 1. The investigators will submit and, where necessary, obtain approval from the above parties for all substantial amendments to the original approved documents.

Dissemination and public engagement

Data from this study will be of interest to the scientific and clinical research communities. An informative resource for managing sepsis will be made available to local clinicians, clinical microbiologists and infection control policy developers. Study data will be reported according to the STROBE guidance for reporting observational studies.(35) The authors (and their respective positions in the author list) will be agreed prior to the start of the study in accordance with the guidelines of the International Committee of Medical Journal Editors. In line with Wellcome Trust policy that the results of publicly-funded research should be freely available, manuscripts arising from this study will be submitted to peer-reviewed journals which enable Open Access. In line with research transparency and greater access to data sharing policy of OUCRU in Vietnam will be implemented. This policy is based on a controlled access approach with a restriction on data release that would compromise an on-going trial or study. Data exchange complies with Information Governance and Data Security Policies in all of the relevant countries.

For peer review only

Discussion

As a conduit for introducing molecular biology for bacteriology into routine hospital care in Vietnam the study is unique, and is planned to lead to future studies investigating appropriate empirical treatment for bacteraemia and the impact of AMR on the outcome of sepsis in the neonatal and paediatric population in Vietnam. By studying and defining disease aetiology, antimicrobial susceptibility patterns and disease outcome we plan to develop an improved approach to managing bloodstream infections in our setting and we will use these data to initiate intervention studies focused on preventing sepsis with AMR pathogens in neonates.

Duration and current status of study

The first patient was recruited in January 2017. At the current time the recruitment is on-going. The expected end date for recruitment is 31 December 2019. We expect to have completed our data analysis plan with a view of results by June 2020.

Authors' contributions

All authors satisfy the criteria for authorship as per the ICMJE 'Recommendations for the Conduct, Reporting, Editing, and Publication of Scholarly Work in Medical Journals'.(36) NDT, TCD, CJB, JIC, AK, EK, LQT, NKM, PTTT, LNTN, NNQM, CNP, NTH, NMX, TCT and SB contributed to the conception and design of the study. NDT, TCD and SB drafted the protocol of the study. This article was drafted by NDT, TCD and SB. CJB, JIC, AK, EK, LQT, NKM, PTTT, LNTN, NNQM, CNP, NTH, NMX, TCT, NDT, TCD and SB read and critically revised the protocol and this article prior to submission.

Funding

This study is supported by the Royal Society and the Wellcome Trust (grant 100087/Z/12/Z). The Oxford University Clinical Research Unit is a Major Overseas Programme funded by the Wellcome Trust (grant 089276/2/09/2). TCD is supported by the National Institutes of Health Research (grant

For peer review only

3557) and supported by a Clinical Lecturer Starter Grant from the Academy of Medical Sciences and Wellcome Trust (grant SGCL015/1005).

Competing interests

All authors have no competing interests to declare.

References

1. Wynn JL, Wong HR, Shanley thomas P, Bizzarro MJ, Saiman L, Polin RA. Time for a neonatal-specific consensus definition for sepsis. Pediatr Crit Care Med [Internet]. 2014;15(6):523–8. Available from: http://www.nature.com/doifinder/10.1038/jp.2015.23%5Cnhttp://cpj.sagepub.com/cgi/doi/10.1177/0009922814553435%5Cnhttp://informahealthcare.com/doi/abs/10.3109/00365548.2014.971053%5Cnhttp://www.ncbi.nlm.nih.gov/pubmed/20876594%5Cnhttp://www.ncbi.nlm.nih.gov/pubmed

2. Bailit JL, Gregory KD, Reddy UM, Gonzalez-Quintero VH, Hibbard JU, Ramirez MM, et al. Maternal and neonatal outcomes by labor onset type and gestational age. Am J Obstet Gynecol. 2010;202(3).

3. Bizzarro MJ, Raskind C, Baltimore RS, Gallagher PG. Seventy-five years of neonatal sepsis at Yale: 1928-2003. Pediatrics [Internet]. 2005;116(3):595–602. Available from: http://pediatrics.aappublications.org/cgi/doi/10.1542/peds.2005-0552

4. Weston EJ, Pondo T, Lewis MM, Martell-Cleary P, Morin C, Jewell B, et al. The burden of invasive early-onset neonatal sepsis in the United States, 2005-2008. Pediatr Infect Dis J [Internet]. 2011;30(11):937–41. Available from: /pmc/articles/PMC3193564/?report=abstract

5. Wortham JM, Hansen NI, Schrag SJ, Hale E, Van Meurs K, Sanchez PJ, et al. Chorioamnionitis and Culture-Confirmed, Early-Onset Neonatal Infections. Pediatrics. 2016;137(1):1–11.

6. Stoll BJ, Hansen N, Fanaroff A a, Wright LL, Carlo W a, Ehrenkranz R a, et al. Late-onset sepsis in very low birth weight neonates: the experience of the NICHD Neonatal Research

For peer review only

Network. Pediatrics. 2002;110(2 Pt 1):285–91.

7. Bizzarro MJ, Jiang Y, Hussain N, Gruen JR, Bhandari V, Zhang H. The Impact of Environmental and Genetic Factors on Neonatal Late-Onset Sepsis. J Pediatr [Internet]. 2011;158(2):234–238.e1. Available from: http://www.sciencedirect.com/science/article/pii/S0022347610006591

8. Rahman S, Hameed A, Roghani MT, Ullah Z, S. R, A. H, et al. Multidrug resistant neonatal sepsis in Peshawar, Pakistan. Arch Dis Child Fetal Neonatal Ed [Internet]. 2002;87(1):F52–4. Available from: http://ovidsp.ovid.com/ovidweb.cgi?T=JS&PAGE=reference&D=emed5&NEWS=N&AN=2002248901%5Cnhttp://ovidsp.ovid.com/ovidweb.cgi?T=JS&PAGE=reference&D=med4&NEWS=N&AN=12091293

9. Huttner A, Harbarth S, Carlet J, Cosgrove S, Goossens H, Holmes A, et al. Antimicrobial resistance: a global view from the 2013 World Healthcare-Associated Infections Forum. Antimicrob Resist Infect Control. 2013 Jan;2:31.

10. Chierakul W, Rajanuwong A, Wuthiekanun V, Teerawattanasook N, Gasiprong M, Simpson A, et al. The changing pattern of bloodstream infections associated with the rise in HIV prevalence in northeastern Thailand. Trans R Soc Trop Med Hyg. 2004 Nov;98(11):678–86.

11. Thi L, Vien M, Baker S, Thao P. High Prevalence of Quinolone Resistance Determinants in Commensal Enterobacteriaceae in Ho Chi Minh City , Viet Nam. Clin Res. :1–27.

12. Polin RA, Saiman L. Nosocomial Infections in the Neonatal Intensive Care Unit. Neoreviews [Internet]. 2003;4(3):e81–9. Available from: http://neoreviews.aappublications.org/content/4/3/e81.abstract

13. Nguyen K Van, Thi Do NT, Chandna A, Nguyen TV, Pham C Van, Doan PM, et al. Antibiotic use and resistance in emerging economies: a situation analysis for Viet Nam. BMC Public Health [Internet]. 2013;13(1):1158. Available from: http://www.biomedcentral.com/1471-2458/13/1158

14. Waters D, Jawad I, Ahmad A, Lukšić I, Nair H, Zgaga L, et al. Aetiology of community-acquired neonatal sepsis in low and middle income countries. J Glob Health [Internet].

For peer review only

2011;1(2):154–70. Available from: http://www.pubmedcentral.nih.gov/articlerender.fcgi?artid=3484773&tool=pmcentrez&rendertype=abstract

15. Chheng K, Carter MJ, Emary K, Chanpheaktra N, Moore CE, Stoesser N, et al. A prospective study of the causes of febrile illness requiring hospitalization in children in Cambodia. PLoS One. 2013 Jan;8(4):e60634.

16. Parry CM, Vinh H, Chinh NT, Wain J, Campbell JI, Hien TT, et al. The influence of reduced susceptibility to fluoroquinolones in Salmonella enterica serovar Typhi on the clinical response to ofloxacin therapy. PLoS Negl Trop Dis. 2011 Jun;5(6):e1163.

17. Nga TVT, Parry CM, Le T, Lan NPH, Diep TS, Campbell JI, et al. The decline of typhoid and the rise of non-typhoid salmonellae and fungal infections in a changing HIV landscape: bloodstream infection trends over 15 years in southern Vietnam. Trans R Soc Trop Med Hyg. 2012 Jan;106(1):26–34.

18. Hoa NTT, Diep TS, Wain J, Parry CM, Hien TT, Smith MD, et al. Community-acquired septicaemia in southern Viet Nam: The importance of multidrug-resistant Salmonella typhi. Trans R Soc Trop Med Hyg. 1998;92(5):503–8.

19. Hao Chung The, Karkey Abhilasha BS. A high-resolution genomic analysis of multi-drug resistant hospital outbreaks of Klebsiella pneumoniae. EMBO Mol Med. 2015;in press.

20. Holt KE, Thieu Nga TV, Thanh DP, Vinh H, Kim DW, Vu Tra MP, et al. Tracking the establishment of local endemic populations of an emergent enteric pathogen. Proc Natl Acad Sci U S A. 2013 Oct;110(43):17522–7.

21. Nguyen TB, Samsura DAA, van der Krabben E, Le A-D. Saigon-Ho Chi Minh City. Cities [Internet]. 2016;50:16–27. Available from: http://linkinghub.elsevier.com/retrieve/pii/S0264275115001183

22. Oeser C, Lutsar I, Metsvaht T, Turner M a, Heath PT, Sharland M. Clinical trials in neonatal sepsis. J Antimicrob Chemother [Internet]. 2013;68(12):2733–45. Available from: http://www.ncbi.nlm.nih.gov/pubmed/23904558

23. Gray JE, Richardson DK, McCormick MC, Workman-Daniels K, Goldmann DA. Neonatal

For peer review only

therapeutic intervention scoring system: a therapy-based severity-of-illness index. Pediatrics [Internet]. 1992;90(4):561–7. Available from: http://www.ncbi.nlm.nih.gov/pubmed/1408510

24. Pérez-pérez FJ, Hanson ND. Detection of Plasmid-Mediated AmpC β -Lactamase Genes in Clinical Isolates by Using Multiplex PCR Downloaded from http://jcm.asm.org/ on September 2 , 2013 by Wellcome Trust Genome Campus Library Detection of Plasmid-Mediated AmpC NL -Lactamase Genes in Cl. 2002;

25. Woodford N, Fagan EJ, Ellington MJ. Multiplex PCR for rapid detection of genes encoding CTX-M extended-spectrum (beta)-lactamases. J Antimicrob Chemother. 2006 Jan;57(1):154–5.

26. Dallenne C, da Costa A, Decr?? D, Favier C, Arlet G. Development of a set of multiplex PCR assays for the detection of genes encoding important ??-lactamases in Enterobacteriaceae. J Antimicrob Chemother. 2010;65(3):490–5.

27. Parry CM, Thuy CT, Dongol S, Karkey A, Vinh H, Chinh NT, et al. Suitable disk antimicrobial susceptibility breakpoints defining Salmonella enterica serovar Typhi isolates with reduced susceptibility to fluoroquinolones. Antimicrob Agents Chemother. 2010 Dec;54(12):5201–8.

28. Jamal W, Rotimi VO, Albert MJ, Khodakhast F, Nordmann P, Poirel L. High prevalence of VIM-4 and NDM-1 metallo-β-lactamase among carbapenem-resistant Enterobacteriaceae. J Med Microbiol. 2013 Aug;62(Pt 8):1239–44.

29. Weidner J, Cassens U, Göhde W, Wüllenweber J, Greve B. A new triplex real time PCR which distinguishes between MRSA, MSSA, and mecA coagulase negative strains by means of melting point analysis using SYTO 9. Clin Lab. 2013 Jan;59(7–8):795–804.

30. Holden MTG, Hsu L-Y, Kurt K, Weinert LA, Mather AE, Harris SR, et al. A genomic portrait of the emergence, evolution, and global spread of a methicillin-resistant Staphylococcus aureus pandemic. Genome Res. 2013 Apr;23(4):653–64.

31. Harris SR, Cartwright EJP, Török ME, Holden MTG, Brown NM, Ogilvy-Stuart AL, et al. Whole-genome sequencing for analysis of an outbreak of meticillin-resistant Staphylococcus

For peer review only

aureus: a descriptive study. Lancet Infect Dis. 2013 Feb;13(2):130–6.

32. Holt KE, Thieu Nga TV, Thanh DP, Vinh H, Kim DW, Vu Tra MP, et al. Tracking the establishment of local endemic populations of an emergent enteric pathogen. Proc Natl Acad Sci U S A [Internet]. 2013;110(43):17522–7. Available from: http://www.ncbi.nlm.nih.gov/pubmed/24082120%5Cnhttp://www.pubmedcentral.nih.gov/articlerender.fcgi?artid=PMC3808646

33. WMA General Assembly. Declaration of Helsinki. Ethical principles for medical research involving human subjects. J Indian Med Assoc [Internet]. 2013;107(6):1–8. Available from: http://www.wma.net/e/policy/b3.htm%5Cnhttp://www.ncbi.nlm.nih.gov/pubmed/24141714

34. ICH Harmonised Tripartite Guideline. ICH harmonized tripartite guideline: Guideline for Good Clinical Practice. J Postgrad Med. 1996;47(1):45–50.

35. NOAH N. The STROBE Initiative STrengthening the Reporting of OBservational studies in Epidemiology (STROBE). Epidemiol Infect [Internet]. 2008;136(7). Available from: http://www.journals.cambridge.org/abstract_S0950268808000733

36. ICMJE. Recommendations for the Conduct, Reporting, Editing, and Publication of Scholarly Work in Medical Journals [Internet]. Citeseer. 2016. p. 1–17. Available from: http://citeseerx.ist.psu.edu/viewdoc/download?doi=10.1.1.408.8144&rep=rep1&type=pdf

For peer review only

Table 1. Clinical and laboratory signs of neonatal sepsis

**Clinical signs of sepsis**	• Abnormal body temperature (core temperature >38.5^0C or <36^0C and/or temperature instability)<br>• Cardiovascular instability (bradycardia [mean heart rate <10th percentile for age in the absence of external vagal stimulus, beta-blockers or congenital heart disease or otherwise unexplained persistent depression over a 0.5–4h time period] or tachycardia [mean heart rate >2SD above normal for age in the absence of external stimulus, chronic unexplained persistent elevation over a 0.5–4h time period] and/or rhythm instability, reduced urinary output (<1 mL/kg/h), hypotension (mean arterial pressure <5th percentile for age), mottled skin, impaired peripheral perfusion)<br>• Respiratory instability (apnea episodes or tachypnea episodes [mean respiratory rate >2SD above normal for age] or increased oxygen requirements or requirement for ventilator support)<br>• Gastrointestinal (feeding intolerance, poor sucking, abdominal distension)<br>• Skin and subcutaneous lesions (petechial rash, sclerema)<br>• Non-specific (irritability, lethargy, hypotonia)
**Laboratory signs of sepsis**	• White blood cells <4×10^9 cells/L or >20×10^9 cells/L<br>• Immature to total neutrophil ratio (I/T) >0.2<br>• Platelet count <100×10^9/L<br>• CRP >15 mg/L or Procalcitonin ≥2 ng/mL<br>• Glucose intolerance (hyperglycemia [blood glucose >180 mg/dl or 10 mmol/L] or hypoglycemia [blood glucose <45 mg/dl or 2.5 mmol/L])<br>• Metabolic acidosis (base excess <–10 mEq/L or serum lactate >2 mmol/L)

For peer review only

Table 2. Study summary

**Title**	The clinical features, antimicrobial susceptibility patterns and genomics of bacteria causing neonatal sepsis in a children's hospital in Vietnam
**Design**	Observational prospective study<br>All organisms isolated from blood will be stored and archived for molecular characterization
**Participants**	Neonates (≤1 month of age) with sepsis
**Planned Enrolment Period**	2017 – 2019
**Primary Objectives**	• To investigate the clinical characteristics of neonatal sepsis.<br>• To define the aetiology, the percentage of positive blood culture and major causes of sepsis.<br>• To investigate the antimicrobial susceptibilities of the pathogens causing sepsis and the rate of antimicrobial resistance.<br>• To measure the impact of sepsis on the severity of disease and the outcomes (mortality rate, length of stay and cost of treatment) of hospitalized neonates.<br>• To analyse the genome sequences of bacterial strains causing neonatal sepsis.
**Secondary Objectives**	• To analyse the antimicrobial resistance profiles and gene distribution by clinical departments to add insight into the circulation of bacteria causing nosocomial infections.<br>• To study the genes catalysing resistance to the antimicrobials commonly used to treat neonatal sepsis (specifically third/fourth generation cephalosporins, fluoroquinolones and carbapenems).

For peer review only

BMJ Open

The clinical features, antimicrobial susceptibility patterns, and genomics of bacteria causing neonatal sepsis in a children's hospital in Vietnam: protocol for a prospective observational study

Journal:	*BMJ Open*
Manuscript ID	bmjopen-2017-019611.R1
Article Type:	Protocol
Date Submitted by the Author:	07-Nov-2017
Complete List of Authors:	Toan, Nguyen; Oxford University Clinical Research Unit (OUCRU), Enteric Group<br>Darton, Thomas; Hospital for Tropical Diseases, Wellcome Trust Major Overseas Programme, Oxford University Clinical Research Unit<br>Boinett, Christine; Hospital for Tropical Diseases, Wellcome Trust Major Overseas Programme, Oxford University Clinical Research Unit<br>Campbell, James; Hospital for Tropical Diseases, Wellcome Trust Major Overseas Programme, Oxford University Clinical Research Unit<br>Karkey, Abhilasha; Hospital for Tropical Diseases, Wellcome Trust Major Overseas Programme, Oxford University Clinical Research Unit<br>Kestelyn, Evelyne; Hospital for Tropical Diseases, Wellcome Trust Major Overseas Programme, Oxford University Clinical Research Unit<br>Thinh, Le; Children's Hospital 1<br>Mau, Nguyen; Children's Hospital 1<br>Tam, Pham Thi Thanh; Children's Hospital 1<br>Nhan, Le Nguyen Thanh; Children's Hospital 1<br>Minh, Ngo Ngoc Quang; Children's Hospital 1<br>Phuong, Cam; Hanh Phuc International Hospital<br>Hung, Nguyen Thanh; Children's hospital one<br>Xuan, Ngo; Pham Ngoc Thach University of Medicine<br>Thuong, Tang; Department of Health<br>Baker, Stephen; Oxford University Clinical Research Unit, Enteric infections
**Primary Subject Heading**:	Paediatrics
Secondary Subject Heading:	Infectious diseases, Genetics and genomics
Keywords:	Sepsis, Neonates, Clinical features, Antimicrobial resistance, Genomics, Outcomes

SCHOLARONE™
Manuscripts

The clinical features, antimicrobial susceptibility patterns, and genomics of bacteria causing neonatal sepsis in a children's hospital in Vietnam: protocol for a prospective observational study

Nguyen Duc Toan [1,2,3,4], Thomas C. Darton [1,5], Christine J. Boinett [1], James I. Campbell [1], Abhilasha Karkey [1], Evelyne Kestelyn [1,2], Le Quoc Thinh [3], Nguyen Kien Mau [3], Pham Thi Thanh Tam [3], Le Nguyen Thanh Nhan [1,3], Ngo Ngoc Quang Minh [3], Cam Ngoc Phuong [6], Nguyen Thanh Hung [3,4], Ngo Minh Xuan [4], Tang Chi Thuong [4,7], and Stephen Baker [1,2,8]

[1] Hospital for Tropical Diseases, Wellcome Trust Major Overseas Programme, Oxford University Clinical Research Unit, Ho Chi Minh City, Vietnam

[2] Centre for Tropical Medicine and Global Health, Nuffield Department of Clinical Medicine, University of Oxford, Oxford, United Kingdom

[3] Children's Hospital 1, Ho Chi Minh City, Vietnam

[4] Pham Ngoc Thach University of Medicine, Ho Chi Minh City, Vietnam

[5] Department of Infection, Immunity and Cardiovascular Disease, University of Sheffield Medical School, Sheffield, United Kingdom

[6] Hanh Phuc International Hospital, Binh Duong Province, Vietnam

[7] Department of Health, Ho Chi Minh City, Vietnam

[8] Department of Medicine, University of Cambridge, Cambridge, United Kingdom

Corresponding author: Professor Stephen Baker, Hospital for Tropical Diseases, 764 Vo Van Kiet, District 5, Ho Chi Minh City, Vietnam. Tel: 84-28-3923 7954. sbaker@oucru.org

Running title: Vietnam neonatal sepsis protocol

For peer review only

Abstract

Introduction: The clinical syndrome of neonatal sepsis, comprising signs of infection, septic shock, and organ dysfunction in infants ≤4weeks of age, is a frequent sequel to bloodstream infection and mandates urgent antimicrobial therapy. Bacterial characterisation and antimicrobial susceptibility testing is vital for ensuring appropriate therapy, as high rates of antimicrobial resistance (AMR), especially in low and middle-income countries (LMICs), may adversely affect outcome. Ho Chi Minh City (HCMC) in Vietnam is a rapidly expanding city in Southeast Asia with a current population of almost 8 million. There are limited contemporary data on the causes of neonatal sepsis in Vietnam, and we hypothesize, that the emergence of multi-drug resistant bacteria is an increasing problem for the appropriate management of sepsis cases. In this study, we aim to investigate the major causes of neonatal sepsis and assess disease outcomes by clinical features, antimicrobial susceptibility profiles, and genome composition.

Method and analysis: We will conduct a prospective observational study to characterize the clinical and microbiological features of neonatal sepsis in a major children's hospital in HCMC. All bacteria isolated from blood subjected to whole genome sequencing. We will compare clinical variables and outcomes between different bacterial species, genome composition and AMR gene content. AMR gene content will be assessed and stratified by species, years, and contributing hospital departments. Genome sequences will be analysed to investigate phylogenetic relationships.

Ethics and dissemination: The study will be conducted in accordance with the principles of the Declaration of Helsinki and the International Council on Harmonization Guidelines for Good Clinical Practice. Ethics approval has been provided by the Oxford Tropical Research Ethics Committee and Children's Hospital 1. The findings will be disseminated at international conferences and peer-reviewed journals.

Trial registration: ISRCTN69124914

Ethics references: Oxford (Oxford Tropical Research Ethics Committee 35-16), Vietnam (Children's Hospital 1 Ethics Committee 73/GCN/BVND1)

For peer review only

Strengths and limitations of this study

- Little is known about the current aetiological agents of neonatal sepsis in Vietnam; this prospective study will integrate clinical assessments with microbiological and detailed whole genome sequence data to characterize the aetiology and outcome of neonatal sepsis in this high mortality setting.
- This study is being performed at the largest secondary/tertiary paediatrics centre in Southern Vietnam; data collection at a single site may limit the applicability to other hospitals in the country.
- Other limitations encountered in designing this study include ethical issues involved in collecting samples from severely ill neonates, lack of current transferable definitions for neonatal sepsis phenotypes, and stochastic variations in numbers of cases recruited due to seasonal variation and continuous changes in community antimicrobial and vaccine use.
- Some contamination of blood cultures is unavoidable in our setting and therefore accurately classifying some isolates as true pathogens in certain cases may be challenging.

For peer review only

Background

Neonatal sepsis is widely recognized as a clinical syndrome of systemic inflammation in response to, or occurring the same time as, a possible or proven infection (frequently by identifying bacterial bloodstream infection) occurring in children ≤28 days of age. A consensus definition of neonatal sepsis has remained a challenge.(1) Globally, the incidence of neonatal sepsis is estimated to be 1–5 cases/1,000 live births but is lower in full-term neonates (1–2 cases/1,000 live births), in whom the incidence is higher in males than females.(2,3) Early-onset sepsis is defined as the start of sepsis symptoms within 72 hours of birth,(1,4) and is often caused by vertical transmission of pathogens during delivery as a result of chorioamnionitis or maternal genital tract colonization.(5) Late-onset sepsis, occurring after 72 hours from birth,(1,6) may be caused by similar vertical transmission or horizontal transmission mechanisms, due to direct contact with the surrounding environment, attendant healthcare staff, or any invasive procedures.(7)

Neonatal sepsis in Southeast Asia

Neonatal sepsis remains a leading cause of neonatal hospital admission, morbidity, and mortality in lower and middle income countries (LMICs).(8) In this setting, bacterial infection, including bacteraemia, is complicated by multidrug resistance, particularly related to healthcare acquired infection, and effective management of neonatal sepsis is increasingly problematic.(8) Recently, the World Health Organization (WHO) has acknowledged the problem of AMR as an endemic and widespread problem in LMICs.(9) In many LMICs untreatable bacterial infections with broadly AMR pathogens are no longer a threat but a common reality. AMR in LMICs represents one of the biggest threats to global health, and are one of the greatest current challenges in infectious disease research.

While AMR is an issue with all types of bacterial infection, the issue is most acute in management of clinical sepsis. This is a particular problem in neonates, due to high mortality/morbidity rates and the timely need for rapid detection and treatment of the causative pathogen. Sepsis demonstrates extensive geographical diversity in both aetiology and proportions of AMR bacteria isolated.(10,11) Understanding the local and regional epidemiology of sepsis in hospitalized neonates is crucial in the

For peer review only

development of rational management and treatment guidelines, especially in high-risk AMR LMICs locations like Vietnam.

Sepsis and AMR in Vietnam

Bacterial sepsis is classified into two major groups according to place of acquisition. Hospital-acquired sepsis is defined in patients with clinical manifestations of sepsis and a confirmatory blood culture collected >48 hours following hospital admission.(12) Hospital-acquired sepsis is a major threat to patient safety, and in locations with poor surveillance and infection control programs such infections are associated with high mortality rates. The incidence of AMR bloodstream infections in Vietnam has increased over recent years, and is predicted to increase further.(13) This trend has comprised an increase in both Gram-negative and Gram-positive pathogens, chiefly *Escherichia coli, Klebsiella pneumoniae, Acinetobacter baumannii, Staphylococcus aureus,* and enterococci.

Community-acquired sepsis is also an important cause of fever among patients admitted to hospitals across South and Southeast Asia. In our practice, community-acquired sepsis is defined as the presence of sepsis with a confirmatory blood culture collected within 48 hours after hospital admission.(14) Distinguishing bacterial infections from other common causes of fever, such as malaria or dengue, can be challenging without diagnostic laboratory support.(15) Important causes of community-acquired bloodstream infection (CA-BSI) in LMICs include *Salmonella* serovars Typhi and Paratyphi A, *E. coli*, *K. pneumoniae*, *S. aureus* and *S. pneumoniae*. We have reported increasing levels of AMR in these common community-acquired pathogens and highlighted the difficulties of accurate diagnosis with traditionally available diagnostics. For example in Nepal and Vietnam antimicrobial resistance in *Salmonella* Typhi and *Salmonella* Paratyphi A has severely restricted the options available for antimicrobial treatment.(16)

The changing aetiology of BSI in Vietnam

The aetiology of CA-BSI in Vietnam has changed considerably over the 20 years. A previous study documented the decline of *Salmonella* Typhi from 2002, the predominant pathogen until this point,

For peer review only

and the subsequent increase in non-typhoidal *Salmonella* and other opportunistic HIV-associated pathogens.(17) This shift is likely to reflect a changing landscape of infectious disease related to the HIV epidemic, urbanization, and secondary social determinants within Vietnam. Vietnam, as with many countries in Asia, is undergoing a rapid economic transition, and programmes to improve sanitary conditions have reduced the overall risk of water-borne infections. HIV-associated opportunistic pathogens have now emerged as the leading cause of bloodstream infections and the primary cause of mortality in hospitalized adult patients in this location. These studies were performed at the Hospital for Tropical diseases in Ho Chi Minh City and thus included mainly adults, including those with HIV, and children but not neonates.(18–20) Therefore, these observations may not be fully representative of the situation in neonates.

Knowledge gaps

Little is known about contemporary antimicrobial susceptibility patterns and their underlying genetic determinants in the major causes of neonatal bacterial of sepsis in Vietnam. Furthermore, the impact of antimicrobial susceptibility and other virulence factors on disease progression and outcome in neonates in LMICs is also not well documented. To address these issues, we aim to investigate the aetiology of pathogens associated with bacterial sepsis in neonates and to detail the effects of reduced antimicrobial susceptibility on the outcome of sepsis in neonates. This will be a clinical and microbiology laboratory research project between Children's Hospital 1 and the Oxford University Clinical Research Unit in Vietnam. This study will be a conduit for introducing molecular biology for bacteriology into routine hospital care at this children's hospital and will lead to future studies investigating appropriate empirical treatment for bacteraemia and the impact of antimicrobial resistance on the outcome of sepsis in the paediatric and neonatal population in Vietnam.

Rationale, aim and objectives

To understand the causes of neonatal sepsis and to best inform antimicrobial treatment regimes in our setting we will perform a prospective observational study at Children's Hospital 1 in Vietnam from 2017 to 2019. There are limited contemporary data on the causes of bacterial sepsis in neonates in

For peer review only

Vietnam. We hypothesize that there have been recent increases in multi-drug resistant Gram-negative bacteria causing sepsis in this high-risk group, and that methicillin-resistant *Staphylococcus aureus* (MRSA) has emerged as an important pathogen. We further aim to investigate the clinical features, major causes of neonatal sepsis, and the distribution of pathogens by departments, their antimicrobial susceptibility patterns and the genomic profiles of the isolated bacteria as well as their association with disease outcomes.

Primary objectives

- To describe the clinical characteristics of neonates with sepsis, including community and hospital-acquired sepsis, early and late-onset sepsis.
- To determine the aetiology of neonatal sepsis and the distribution of pathogens by clinical departments including the Neonatology Department and the Neonatal Intensive Care Unit.
- To determine the antimicrobial susceptibility profiles of the bacteria causing neonatal sepsis and the AMR profiles occurring in community and hospital-acquired infections.
- To analyse the impact of specific bacteria and AMR profile on the outcomes (mortality, length of stay and cost of treatment) of neonates with sepsis.
- To determine the genome sequences of bacterial strains associated with neonatal sepsis.

Secondary objectives

- To determine the AMR profiles and gene distribution of isolated bacteria by clinical departments to add insight into the circulation of bacteria associated with hospital acquired infections.
- To study the genes catalysing resistance to antimicrobials commonly used to treat neonatal sepsis (specifically third/fourth generation cephalosporins, fluoroquinolones and carbapenems).

For peer review only

Methods

Study design

This protocol describes a prospective, non-interventional, observational study to characterize the clinical features of neonates with sepsis at Children’s Hospital 1 in Ho Chi Minh City in Vietnam between 2017 and 2019, the microbial population structure, antimicrobial susceptibility patterns and the AMR genes of the bacteria causing that sepsis. All organisms isolated from blood will be stored and archived for molecular characterization.

Study site

Children’s Hospital 1 (Neonatology Department, Neonatal Intensive Care Unit, Microbiology Department) is in Ho Chi Minh City in Vietnam. The estimated population of the city was 8.4 million in 2016, and 23.8% are children 0-14 years of age.(21) Children's Hospital 1 is the largest tertiary paediatrics centre in Southern Vietnam with 1,400 inpatient beds and >1,600 staff members. The hospital receives ~1.5 million outpatient visits and 95,000 admissions each year. Care is provided to all children <15 years old from Ho Chi Minh City and other provinces of Southern Vietnam. The neonatal centre at this hospital currently has 120 inpatient beds for the neonatology department and additional 30 beds in the neonatal intensive care unit. The overall mean rate of positive blood cultures in our hospital is 7% per year.

Definitions

Definition of sepsis

A sepsis episode in this study is defined as isolation of a clinically relevant pathogen from ≥1 blood culture, drawn from a neonate with ≥1 clinical or laboratory sign of sepsis (Table 1).(22)

Diagnosis of neonatal sepsis

Systematic guidelines concerning which patients should have blood cultures performed are not strictly defined in our hospital, although blood culture results are used to confirm the diagnosis of sepsis in neonates with a compatible clinical presentation. We use the criteria suggested by the European

For peer review only

Medicines Agency (EMA) in 2010 for the diagnosis of "probable sepsis" and "confirmed sepsis" in neonates:(22)

- Probable sepsis: ≥2 clinical and ≥2 laboratory signs.
- Confirmed sepsis: ≥1 positive culture of a pathogen and ≥1 clinical or laboratory sign.

Sample size

In this prospective observational study, we aim to recruit all patients with available data who fulfil the inclusion criteria and are admitted to Children's Hospital 1 in Ho Chi Minh City in Vietnam from 2017 – 2019. Based on retrospective surveillance data, we estimate recruitment of 800 participants during the study period. Blood cultures will be performed in all cases, we expect to yield ~400 bacterial isolates.

Participant selection and recruitment

Inclusion criteria

Neonates (≤1 month of age) with a diagnosis of "probable" or "confirmed" sepsis who have had a blood culture taken and who are an in-patient at Children's Hospital 1 will be recruited into the study, after written informed consent has been given by a parent or guardian.

Exclusion criteria

Patients will be excluded when informed consent is not provided, the length of hospital stay less than 24 hours, imminent and inevitable death, or the patient has been previously recruited in the study. Investigators will review all of the mortality records during the time period of the study to try and identify how many of these cases may have been missed and whether there were any common characteristics in these participants.

Identification of participants

All doctors and nurses in the Department of Neonatology and Neonatal Intensive Care Unit of the study hospital will be informed about, and trained for, this clinical investigation. In addition, those working in the Department of Neonatology and Neonatal Intensive Care Unit will also be involved in the study. These staff will be trained to identify eligible patients and how to notify investigators.

For peer review only

Informed consent

Trained, GCP-accredited, members of the study team will collect informed consent. The team will discuss the study with the accompanying parent/guardian, or, if both parents are deceased or not actively involved in child care, the main long-term carer of the child will be accepted as the guardian and considered able to give consent for the study. Study staff will describe the purpose of the study, the study procedures, possible risks/benefits, the rights and responsibilities of participants, and alternatives to enrolment. The parent/guardian will be invited to ask questions, which will be addressed by study staff, and they will be provided with appropriate contact numbers if they have any subsequent questions. If the parent/guardian agrees for the child to participate, they will be asked to sign and date an informed consent form. A copy of the patient information sheet and the informed consent form will be given to them to keep. In addition to the procedures above, illiterate signatories will have the Informed Consent Form read to them in the presence of a witness who will sign to confirm this. The parent/guardian can withdraw from the study at any time (verbally) without affecting the care that the child will receive. If the parent/guardian decides at any time to take the child out of the study, no new information will be collected. However, information collected on the child up until that point will still be used. All patient information sheets and consent forms will be written in the local language and will use terms that are easily understandable.

Study procedures

An investigator will routinely record and collect demographic, clinical and laboratory information of the patients, the date of blood draw, the number of blood culture bottles inoculated, the result of the culture (whether positive or negative) and the susceptibility of the isolate to commonly used antimicrobials. Data from these records will be subsequently entered into CliRes Data Management System of Oxford University Clinical Research Unit. These will be source data for this study. The number of patients admitted to the hospital annually will be obtained from hospital records. As part of this study we request that all isolates from blood are stored and archived at –80^{0}C. These isolates will

For peer review only

be re-cultured and the identification will be re-confirmed. Selected isolated organisms from blood will have further molecular characterization at a later date.

Data collection

Demographic and clinical assessments

Data on neonatal sepsis at Children's Hospital 1 will be collected to the case report form. These data will include administrative data, demographic data, clinical characteristics, laboratory results, diagnoses, treatments and outcomes. The Neonatal Therapeutic Intervention Scoring System (NTISS) will be used to estimate the disease severity.(23)

Microbiological assessments

Available routine microbiology data on neonatal bloodstream infections Children's Hospital 1. These data will include pathogenic agents isolated from blood culture and antimicrobial susceptibility profile of isolated bacteria (routine panel of antimicrobials). Selected isolates will have additional antimicrobial susceptibility testing, molecular analysis for antimicrobial resistance genes and genome sequencing of selected strains defined by antimicrobial susceptibility data.

Laboratory methods

Microbiology testing

When required for checking or to non-routine antimicrobials, antimicrobial susceptibility testing of the pathogens isolated will be performed by disk diffusion using guidelines established by the Clinical and Laboratory Standards Institute (CLSI) and, when required, by minimum inhibitory concentration estimation (MIC) using the VITEK 2 COMPACT automated machine. Antimicrobial susceptibilities tested will include nalidixic acid, ciprofloxacin, ceftriaxone, cefepime, ampicillin, trimethoprim-sulfamethoxazole, azithromycin, imipenem, colistin and amikacin for all Gram-negative organisms and oxacillin and vancomycin in Gram-positive organisms. The production of extended-spectrum beta lactamases (ESBL) will be investigated using the double-disc synergy test by comparing zone sizes between ceftazidime discs against ceftazidime-clavulanic acid discs and cefotaxime discs against

For peer review only

cefotaxime-clavulanic acid discs. Isolates with an increase in diameter of inhibitory zone of equal to or more than 5 mm by the synergy of clavulanate will be considered ESBL positive.

Organisms including Coryneforms (*Corynebacterium*, etc.), *Micrococci*, *Propionibacterium*, *Bacillus*, alpha hemolytic *Streptococci*, environmental Gram-negative bacilli, and non-pathogenic *Neisseria* will be considered potential contaminants. The pathogen-contaminant decision will be made based on the clinical relevance of the isolated bacteria and the independent assessments by two qualified medical microbiologists. If there is disagreement then the case will be discussed until a decision is reached.

Bacterial storage

Organisms will be sub-cultured onto 5% blood agar and the purity of the isolate will be tested before storage in 20% glycerol at –80°C.

Isolation of nucleic acids

Isolates will be re-cultured and their identification re-checked. DNA will be extracted from bacterial isolates using the Wizard Genomic DNA Extraction Kit (Promega, Fitchburg, USA). The quality and concentration of the DNA will be assessed using a nano-drop spectrophotometer prior to PCR amplification and the Quant-IT Kit (Invitrogen, Carlsbad, CA) prior to DNA sequencing.

PCR for resistance genes

The primary focus study is to investigate the distribution of antimicrobial resistance genes in bacteria causing neonatal sepsis. Therefore, all Gram-negative organisms will be investigated by PCR to detect genes catalyzing resistance to cephalosporins, fluoroquinolones and carbapenems. Conventional PCR will be performed for the following classes of resistance genes using previously described methods. The multiplex and monoplex PCRs are described in these publications. PCR will be used to detect; AmpC, ESBL (including CTX-M15), NDM, qnr, OXA, KPC and mecA/Van.(11,24–29)

For peer review only

Genome sequencing

Selected organisms (on the basis of their susceptibility profiles and resistance gene content) will be genome sequenced. We aim to sequence the greatest cross-section of organism groups as possible (i.e. all *Staphylococci* or all *Klebsiella*). Selected bacterial isolates will be sequenced at Oxford University Clinical Research Unit in Vietnam. Briefly, index-tagged paired end Illumina sequencing libraries will be prepared using one of 96 unique indexing tags as previously described. These will be combined into pools of uniquely tagged libraries and sequenced on the Illumina Genome Analyzer or HiSeq sequencer according to manufacturer's protocols to generate tagged 54-100 bp paired-end reads. This is previously approached for describing Gram-negative organisms and *Staphylococcus*.(20,30,31)

Analysis plan

Statistical comparisons

Data will be presented in the form of tables and bar charts for descriptive variables i.e. number of organisms per year and number of AMR organisms per year. Statistical comparisons of features between groups (positive/negative blood culture, Gram negative/Gram positive bacteria, survival/non-survival etc.) and time trend analysis of the cultured isolates by month and the antimicrobial susceptibility patterns will be conducted. These data will be placed in the context of the broader population, by comparison of these data with historical laboratory records of pathogens isolated from patients with bloodstream infections. Historical data from both neonates and older children will be analysed descriptively, and where appropriate, time trend analyses will be performed to determine significant alterations in bloodstream infection aetiology. All statistical analysis will be performed using Stata version 14 (Stata Corp LP, College Station, TX, USA) and R. P-values of ≤ 0.05 will be considered significant.

Antimicrobial resistance genes and genome sequencing

The presence/absence of antimicrobial resistance genes will be reported as proportions per organism and then stratified by organism, year, and hospital department. Genome sequences will be determined

For peer review only

to study phylogenetic relationships, the presence/absence of virulence genes and also AMR gene content and firstly analysed by species and then group by their Gram-stain results. Briefly, for phylogenetic analysis, chromosomal Single Nucleotide Polymorphism (SNP) alleles will be concatenated for each strain to generate a multiple alignment of all SNPs. For maximum likelihood (ML) analysis, RAxML will be run using the generalized time-reversible model and one thousand bootstrap pseudo-replicate analyses were performed to assess support for the ML phylogeny. Root-to-tip branches will be extracted from the ML tree using the program TreeStat. The relationship between root-to-tip distances and year of isolation will be analyzed using linear regression. For BEAST analysis (v1.6), a GTR+Γ substitution model and defined tip dates, as the date of isolation will be used.(30–32) To detect the presence or absence of genes read sets will be assembled using the *de novo* short read assembler Velvet and Velvet Optimizer. Organism specific read sets will then be aligned to the pan-genome. Taxonomic investigation of accessory and AMR genes will be performed using MG-RAST v3.2.

Ethics, regulatory approvals and governance

This study is sponsored by the University of Oxford and will be monitored by the Clinical Trials Unit at Oxford University Clinical Research Unit (OUCRU). The Principal Investigator (SB) will ensure that this study is conducted in accordance with the principles of the Declaration of Helsinki and the terms of approval of the appropriate ethical committees.(33) The study will be conducted in full conformity with relevant regulations and with the International Council on Harmonization (ICH) Guidelines for Good Clinical Practice (GCP).(34) This protocol and the relevant supporting document have already had the approvals of the Oxford Tropical Research Ethics Committee (OxTREC) and the institutional review board (IRB) of Children's Hospital 1. The investigators will submit and, where necessary, obtain approval from the above parties for all substantial amendments to the original approved documents.

Dissemination and public engagement

For peer review only

Data from this study will be of interest to the scientific and clinical research communities. An informative resource for managing sepsis will be made available to local clinicians, clinical microbiologists and infection control policy developers. Study data will be reported according to the STROBE guidance for reporting observational studies.(35) The authors (and their respective positions in the author list) will be agreed prior to the start of the study in accordance with the guidelines of the International Committee of Medical Journal Editors. In line with Wellcome Trust policy that the results of publicly-funded research should be freely available, manuscripts arising from this study will be submitted to peer-reviewed journals which enable Open Access. In line with research transparency and greater access to data sharing policy of OUCRU in Vietnam will be implemented. This policy is based on a controlled access approach with a restriction on data release that would compromise an on-going trial or study. Data exchange complies with Information Governance and Data Security Policies in all of the relevant countries.

Discussion

As a conduit for introducing molecular biology for bacteriology into routine hospital care in Vietnam the study is unique, and is planned to lead to future studies investigating appropriate empirical treatment for bacteraemia and the impact of AMR on the outcome of sepsis in the neonatal and paediatric population in Vietnam. By studying and defining disease aetiology, antimicrobial susceptibility patterns and disease outcome we plan to develop an improved approach to managing bloodstream infections in our setting and we will use these data to initiate intervention studies focused on preventing sepsis with AMR pathogens in neonates. Table 2 shows the summary of this study.

Duration and current status of study

The first patient was recruited in January 2017. At the current time the recruitment is on-going. The expected end date for recruitment is 31 December 2019. We expect to have completed our data analysis plan with a view of results by June 2020.

Authors' contributions

For peer review only

All authors satisfy the criteria for authorship as per the ICMJE 'Recommendations for the Conduct, Reporting, Editing, and Publication of Scholarly Work in Medical Journals'. NDT, TCD, CJB, JIC, AK, EK, LQT, NKM, PTTT, LNTN, NNQM, CNP, NTH, NMX, TCT and SB contributed to the conception and design of the study. NDT, TCD and SB drafted the protocol of the study. This article was drafted by NDT, TCD and SB. CJB, JIC, AK, EK, LQT, NKM, PTTT, LNTN, NNQM, CNP, NTH, NMX, TCT, NDT, TCD and SB read and critically revised the protocol and this article prior to submission.

Funding

This study is supported by the Royal Society and the Wellcome Trust (grant 100087/Z/12/Z). The Oxford University Clinical Research Unit is a Major Overseas Programme funded by the Wellcome Trust (grant 089276/2/09/2). TCD is supported by the National Institutes of Health Research (grant 3557) and supported by a Clinical Lecturer Starter Grant from the Academy of Medical Sciences and Wellcome Trust (grant SGCL015/1005).

Competing interests

All authors have no competing interests to declare.

References

1. Wynn JL, Wong HR, Shanley thomas P, Bizzarro MJ, Saiman L, Polin RA. Time for a neonatal-specific consensus definition for sepsis. Pediatr Crit Care Med [Internet]. 2014;15(6):523–8. Available from: http://www.nature.com/doifinder/10.1038/jp.2015.23%5Cnhttp://cpj.sagepub.com/cgi/doi/10.1177/0009922814553435%5Cnhttp://informahealthcare.com/doi/abs/10.3109/00365548.2014.971053%5Cnhttp://www.ncbi.nlm.nih.gov/pubmed/20876594%5Cnhttp://www.ncbi.nlm.nih.gov/pubmed

2. Bailit JL, Gregory KD, Reddy UM, Gonzalez-Quintero VH, Hibbard JU, Ramirez MM, et al. Maternal and neonatal outcomes by labor onset type and gestational age. Am J Obstet

For peer review only

Gynecol. 2010;202(3).

3. Bizzarro MJ, Raskind C, Baltimore RS, Gallagher PG. Seventy-five years of neonatal sepsis at Yale: 1928-2003. Pediatrics [Internet]. 2005;116(3):595–602. Available from: http://pediatrics.aappublications.org/cgi/doi/10.1542/peds.2005-0552

4. Weston EJ, Pondo T, Lewis MM, Martell-Cleary P, Morin C, Jewell B, et al. The burden of invasive early-onset neonatal sepsis in the United States, 2005-2008. Pediatr Infect Dis J [Internet]. 2011;30(11):937–41. Available from: /pmc/articles/PMC3193564/?report=abstract

5. Wortham JM, Hansen NI, Schrag SJ, Hale E, Van Meurs K, Sanchez PJ, et al. Chorioamnionitis and Culture-Confirmed, Early-Onset Neonatal Infections. Pediatrics. 2016;137(1):1–11.

6. Stoll BJ, Hansen N, Fanaroff A a, Wright LL, Carlo W a, Ehrenkranz R a, et al. Late-onset sepsis in very low birth weight neonates: the experience of the NICHD Neonatal Research Network. Pediatrics. 2002;110(2 Pt 1):285–91.

7. Bizzarro MJ, Jiang Y, Hussain N, Gruen JR, Bhandari V, Zhang H. The Impact of Environmental and Genetic Factors on Neonatal Late-Onset Sepsis. J Pediatr [Internet]. 2011;158(2):234–238.e1. Available from: http://www.sciencedirect.com/science/article/pii/S0022347610006591

8. Rahman S, Hameed A, Roghani MT, Ullah Z, S. R, A. H, et al. Multidrug resistant neonatal sepsis in Peshawar, Pakistan. Arch Dis Child Fetal Neonatal Ed [Internet]. 2002;87(1):F52–4. Available from: http://ovidsp.ovid.com/ovidweb.cgi?T=JS&PAGE=reference&D=emed5&NEWS=N&AN=2002248901%5Cnhttp://ovidsp.ovid.com/ovidweb.cgi?T=JS&PAGE=reference&D=med4&NEWS=N&AN=12091293

9. Huttner A, Harbarth S, Carlet J, Cosgrove S, Goossens H, Holmes A, et al. Antimicrobial resistance: a global view from the 2013 World Healthcare-Associated Infections Forum. Antimicrob Resist Infect Control. 2013 Jan;2:31.

10. Chierakul W, Rajanuwong A, Wuthiekanun V, Teerawattanasook N, Gasiprong M, Simpson A, et al. The changing pattern of bloodstream infections associated with the rise in HIV

For peer review only

prevalence in northeastern Thailand. Trans R Soc Trop Med Hyg. 2004 Nov;98(11):678–86.

11. Thi L, Vien M, Baker S, Thao P. High Prevalence of Quinolone Resistance Determinants in Commensal Enterobacteriaceae in Ho Chi Minh City , Viet Nam. Clin Res. :1–27.

12. Polin RA, Saiman L. Nosocomial Infections in the Neonatal Intensive Care Unit. Neoreviews [Internet]. 2003;4(3):e81–9. Available from: http://neoreviews.aappublications.org/content/4/3/e81.abstract

13. Nguyen K Van, Thi Do NT, Chandna A, Nguyen TV, Pham C Van, Doan PM, et al. Antibiotic use and resistance in emerging economies: a situation analysis for Viet Nam. BMC Public Health [Internet]. 2013;13(1):1158. Available from: http://www.biomedcentral.com/1471-2458/13/1158

14. Waters D, Jawad I, Ahmad A, Lukšić I, Nair H, Zgaga L, et al. Aetiology of community-acquired neonatal sepsis in low and middle income countries. J Glob Health [Internet]. 2011;1(2):154–70. Available from: http://www.pubmedcentral.nih.gov/articlerender.fcgi?artid=3484773&tool=pmcentrez&rendertype=abstract

15. Chheng K, Carter MJ, Emary K, Chanpheaktra N, Moore CE, Stoesser N, et al. A prospective study of the causes of febrile illness requiring hospitalization in children in Cambodia. PLoS One. 2013 Jan;8(4):e60634.

16. Parry CM, Vinh H, Chinh NT, Wain J, Campbell JI, Hien TT, et al. The influence of reduced susceptibility to fluoroquinolones in Salmonella enterica serovar Typhi on the clinical response to ofloxacin therapy. PLoS Negl Trop Dis. 2011 Jun;5(6):e1163.

17. Nga TVT, Parry CM, Le T, Lan NPH, Diep TS, Campbell JI, et al. The decline of typhoid and the rise of non-typhoid salmonellae and fungal infections in a changing HIV landscape: bloodstream infection trends over 15 years in southern Vietnam. Trans R Soc Trop Med Hyg. 2012 Jan;106(1):26–34.

18. Hoa NTT, Diep TS, Wain J, Parry CM, Hien TT, Smith MD, et al. Community-acquired septicaemia in southern Viet Nam: The importance of multidrug-resistant Salmonella typhi. Trans R Soc Trop Med Hyg. 1998;92(5):503–8.

For peer review only

19. Hao Chung The, Karkey Abhilasha BS. A high-resolution genomic analysis of multi-drug resistant hospital outbreaks of Klebsiella pneumoniae. EMBO Mol Med. 2015;in press.

20. Holt KE, Thieu Nga TV, Thanh DP, Vinh H, Kim DW, Vu Tra MP, et al. Tracking the establishment of local endemic populations of an emergent enteric pathogen. Proc Natl Acad Sci U S A. 2013 Oct;110(43):17522–7.

21. Nguyen TB, Samsura DAA, van der Krabben E, Le A-D. Saigon-Ho Chi Minh City. Cities [Internet]. 2016;50:16–27. Available from: http://linkinghub.elsevier.com/retrieve/pii/S0264275115001183

22. Oeser C, Lutsar I, Metsvaht T, Turner M a, Heath PT, Sharland M. Clinical trials in neonatal sepsis. J Antimicrob Chemother [Internet]. 2013;68(12):2733–45. Available from: http://www.ncbi.nlm.nih.gov/pubmed/23904558

23. Gray JE, Richardson DK, McCormick MC, Workman-Daniels K, Goldmann DA. Neonatal therapeutic intervention scoring system: a therapy-based severity-of-illness index. Pediatrics [Internet]. 1992;90(4):561–7. Available from: http://www.ncbi.nlm.nih.gov/pubmed/1408510

24. Pérez-pérez FJ, Hanson ND. Detection of Plasmid-Mediated AmpC β -Lactamase Genes in Clinical Isolates by Using Multiplex PCR Downloaded from http://jcm.asm.org/ on September 2 , 2013 by Wellcome Trust Genome Campus Library Detection of Plasmid-Mediated AmpC NL -Lactamase Genes in Cl. 2002;

25. Woodford N, Fagan EJ, Ellington MJ. Multiplex PCR for rapid detection of genes encoding CTX-M extended-spectrum (beta)-lactamases. J Antimicrob Chemother. 2006 Jan;57(1):154–5.

26. Dallenne C, da Costa A, Decr?? D, Favier C, Arlet G. Development of a set of multiplex PCR assays for the detection of genes encoding important ??-lactamases in Enterobacteriaceae. J Antimicrob Chemother. 2010;65(3):490–5.

27. Parry CM, Thuy CT, Dongol S, Karkey A, Vinh H, Chinh NT, et al. Suitable disk antimicrobial susceptibility breakpoints defining Salmonella enterica serovar Typhi isolates with reduced susceptibility to fluoroquinolones. Antimicrob Agents Chemother. 2010

For peer review only

Dec;54(12):5201–8.

28. Jamal W, Rotimi VO, Albert MJ, Khodakhast F, Nordmann P, Poirel L. High prevalence of VIM-4 and NDM-1 metallo-β-lactamase among carbapenem-resistant Enterobacteriaceae. J Med Microbiol. 2013 Aug;62(Pt 8):1239–44.

29. Weidner J, Cassens U, Göhde W, Wüllenweber J, Greve B. A new triplex real time PCR which distinguishes between MRSA, MSSA, and mecA coagulase negative strains by means of melting point analysis using SYTO 9. Clin Lab. 2013 Jan;59(7–8):795–804.

30. Holden MTG, Hsu L-Y, Kurt K, Weinert LA, Mather AE, Harris SR, et al. A genomic portrait of the emergence, evolution, and global spread of a methicillin-resistant Staphylococcus aureus pandemic. Genome Res. 2013 Apr;23(4):653–64.

31. Harris SR, Cartwright EJP, Török ME, Holden MTG, Brown NM, Ogilvy-Stuart AL, et al. Whole-genome sequencing for analysis of an outbreak of meticillin-resistant Staphylococcus aureus: a descriptive study. Lancet Infect Dis. 2013 Feb;13(2):130–6.

32. Holt KE, Thieu Nga TV, Thanh DP, Vinh H, Kim DW, Vu Tra MP, et al. Tracking the establishment of local endemic populations of an emergent enteric pathogen. Proc Natl Acad Sci U S A [Internet]. 2013;110(43):17522–7. Available from: http://www.ncbi.nlm.nih.gov/pubmed/24082120%5Cnhttp://www.pubmedcentral.nih.gov/articlerender.fcgi?artid=PMC3808646

33. WMA General Assembly. Declaration of Helsinki. Ethical principles for medical research involving human subjects. J Indian Med Assoc [Internet]. 2013;107(6):1–8. Available from: http://www.wma.net/e/policy/b3.htm%5Cnhttp://www.ncbi.nlm.nih.gov/pubmed/24141714

34. ICH Harmonised Tripartite Guideline. ICH harmonized tripartite guideline: Guideline for Good Clinical Practice. J Postgrad Med. 1996;47(1):45–50.

35. NOAH N. The STROBE Initiative STrengthening the Reporting of OBservational studies in Epidemiology (STROBE). Epidemiol Infect [Internet]. 2008;136(7). Available from: http://www.journals.cambridge.org/abstract_S0950268808000733

For peer review only

Table 1. Clinical and laboratory signs of neonatal sepsis

**Clinical signs of sepsis**	• Abnormal body temperature (core temperature >38.5^{0}C or <36^{0}C and/or temperature instability)<br>• Cardiovascular instability (bradycardia [mean heart rate <10th percentile for age in the absence of external vagal stimulus, beta-blockers or congenital heart disease or otherwise unexplained persistent depression over a 0.5–4h time period] or tachycardia [mean heart rate >2SD above normal for age in the absence of external stimulus, chronic unexplained persistent elevation over a 0.5–4h time period] and/or rhythm instability, reduced urinary output (<1 mL/kg/h), hypotension (mean arterial pressure <5th percentile for age), mottled skin, impaired peripheral perfusion)<br>• Respiratory instability (apnea episodes or tachypnea episodes [mean respiratory rate >2SD above normal for age] or increased oxygen requirements or requirement for ventilator support)<br>• Gastrointestinal (feeding intolerance, poor sucking, abdominal distension)<br>• Skin and subcutaneous lesions (petechial rash, sclerema)<br>• Non-specific (irritability, lethargy, hypotonia)
**Laboratory signs of sepsis**	• White blood cells <4×10^9 cells/L or >20×10^9 cells/L<br>• Immature to total neutrophil ratio (I/T) >0.2<br>• Platelet count <100×10^9/L<br>• CRP >15 mg/L or Procalcitonin ≥2 ng/mL<br>• Glucose intolerance (hyperglycemia [blood glucose >180 mg/dl or 10 mmol/L] or hypoglycemia [blood glucose <45 mg/dl or 2.5 mmol/L])<br>• Metabolic acidosis (base excess <–10 mEq/L or serum lactate >2 mmol/L)

For peer review only

Table 2. Study summary

**Title**	The clinical features, antimicrobial susceptibility patterns and genomics of bacteria causing neonatal sepsis in a children's hospital in Vietnam
**Design**	Observational prospective study<br>All organisms isolated from blood will be stored and archived for molecular characterization
**Participants**	Neonates (≤1 month of age) with sepsis
**Planned Enrolment Period**	2017 – 2019
**Primary Objectives**	• To investigate the clinical characteristics of neonatal sepsis.<br>• To define the aetiology, the percentage of positive blood culture and major causes of sepsis.<br>• To investigate the antimicrobial susceptibilities of the pathogens causing sepsis and the rate of antimicrobial resistance.<br>• To measure the impact of sepsis on the severity of disease and the outcomes (mortality rate, length of stay and cost of treatment) of hospitalized neonates.<br>• To analyse the genome sequences of bacterial strains causing neonatal sepsis.
**Secondary Objectives**	• To analyse the antimicrobial resistance profiles and gene distribution by clinical departments to add insight into the circulation of bacteria causing nosocomial infections.<br>• To study the genes catalysing resistance to the antimicrobials commonly used to treat neonatal sepsis (specifically third/fourth generation cephalosporins, fluoroquinolones and carbapenems).

For peer review only
